# Joint monitoring of mean and variance using Max-EWMA control chart under lognormal process with application to engine oil data

**DOI:** 10.1038/s41598-024-64292-1

**Published:** 2024-06-15

**Authors:** Fatimah A. Almulhim, Seher Malik, Muhammad Hanif, Abaker A. Hassaballa, Muhammad Nabi, Muhammad Usman Aslam

**Affiliations:** 1 Department of Mathematical Sciences, College of Science, Princess Nourah Bint Abdulrahman University, P.O. Box 84428, 11671 Riyadh, Saudi Arabia; 2 National College of Business Administration and Economics, Lahore, Pakistan; 3 Department of Mathematics, Faculty of Science, Northern Border University, Arar, Saudi Arabia; 4 Khost Mechanics Institute, Khost, Afghanistan; 5 Xi’an Jiaotong University, Xi’an, China

**Keywords:** Max-EWMA, Lognormal, ARL, SDRL, Control charts, Engineering, Materials science

## Abstract

The control charts are frequently employed in process monitoring to assess the average and variability of a process, assuming a normal distribution. However, it is worth noting that some process distributions tend to exhibit a positively skewed distribution, such as the lognormal distribution. This article proposed a maximum exponential weighted moving average control chart for joint monitoring of mean and variance under a lognormal process. The proposed control chart is evaluated by using the run length profile such as ARL and SDRL. The Monte Carlo simulation is conducted by using the R language to find the run length profile. An application is presented to demonstrate the design of the proposed control chart.

## Introduction

There have been advancements in the implementation of statistical process control mechanisms to monitor production processes. These mechanisms aim to prevent errors and defective products by promptly identifying the underlying reasons for any unexpected fluctuations. The statistical process control uses control charts as one of its instruments for monitoring processes for alterations. A control chart is the most crucial tool for monitoring processes and discovering faults in this context. The prompt detection of shifts has proven to be effective in mitigating company losses. A control chart serves as a valuable tool for process monitoring and enables them to make timely and accurate decisions to restore production to a stable state. The well-known of these control charts is Shewhart control chart which was designed to keep the mean and dispersion under control. When it comes to spotting large changes in the process, Shewhart control charts perform admirably. However, their performance was inadequate for small to moderate changes. Because of this, in 1954, Page^1^ developed the CUSUM control chart, which is useful for spotting small changes in the process. Control charts have been widely utilized in various industries, particularly for monitoring processes that follow a normal distribution. However, it is important to note that a given process may deviate from the normal distribution and exhibit positive or right skewness. In a study conducted by Morrison^2^, as well as Joffe and Sichel^3^ and Kotz and Lovelace^4^ presented illustrative situations of processes that adhere to the lognormal distribution.

The evaluation of a control chart's performance is commonly conducted by an analysis of the run length distribution of the control chart. The run length (RL) of a control chart is formally characterized as the quantity of samples acquired prior to the occurrence of an out-of-control signal. Similarly, the average run length (ARL) denotes the anticipated value of the run length. Numerous research has elucidated the impact of parameter estimates on the efficacy of control charts. For instance, refer to the works of Chen^5^, Quesenberry^6^, Jones et al.^7^, Bischak and Trietsch^8^, Castagliola et al.^9^, Jensen et al.^10^ and Psarakis et al.^11^. They have provided a thorough examination of the current amount of literature about the influence of parameter estimates on the efficacy of control charts. Various research has been conducted in the literature about the development of control charts to monitor the mean of a lognormal distribution. Morrison^2^ introduced a revised control charting method that employed the sample ratio. Ferrell^12^ introduced a control chart that was used to monitor the geometric midrange of a lognormal distribution. In their study, Joffe and Sichel^3^ developed a control chart to effectively monitor the arithmetic mean of a lognormal distribution. Shaheen et al.^13^ introduced a monitoring control chart that utilizes a repeating sampling strategy to account for lognormal process variance. In their study, Omar et al.^14^ introduced a highly effective methodology for the surveillance of a process that exhibits a positive skew. Control charts for simultaneous mean and variance monitoring were discussed in detail by McCracken and Chakraborti^15^, Yang^16^ advocated using a single-average loss control chart to keep an eye on the mean and standard deviation of a process. This study focuses on the development of three integrated X and S-charts for simultaneous monitoring of the mean and standard deviation of the lognormal distribution. The results of the simulation study indicate that the combined lognormal X and S-charts exhibit favorable performance in cases where the underlying lognormal distribution is highly skewed. Figueiredo and Gomes^17^, Adekeye and Azubuike^18^ focused on the development of resilient control charts specifically tailored for non-normal processes. Huang et al.^19^ introduced a lognormal S control chart to monitor the lognormal standard deviation. Their findings indicated that the proposed S-chart exhibited superior performance as compared to alternative methods. Akhtar et al.^20^ developed an EWMA chart customized for the lognormal process. Iqbal et al.^21^ introduced a control chart for monitoring the process mean and variability under the Bayesian Approach. The suggested method utilized Max-EWMA statistic for joint monitoring with different loss functions. Huang^22^ conducted a study on the lognormal process by employing separate $\overline{X }$ and S-charts to simultaneously monitor the lognormal mean and standard deviation. In this study, we built upon the previous research conducted by Huang^22^ and introduced a novel methodology for simultaneously monitoring the scale and shape parameters in a lognormal distribution process with a single statistic.

While existing literature predominantly explores joint monitoring control charts for mean and variance in normal distributions, our contribution fills a notable gap by addressing the complexities of non-normal distributions. This study advances the understanding and application of joint monitoring control charts within the context of non-normal distributions, specifically focusing on the lognormal distribution. This study introduces a methodology that extends the capabilities of traditional control charts to simultaneously monitor the scale and shape parameters inherent in lognormal processes. This research not only expands the theoretical framework of the joint monitoring control chart but also provides practical application where lognormal distribution is prevalent. By offering a single statistic solution for monitoring scale and shape parameters, our methodology enhances the efficiency and accuracy of quality control processes in lognormal distribution scenarios.

The subsequent sections of the paper are structured in the following manner. Section "Design of Proposed Control Chart" provides a discussion on both the lognormal distribution and the proposed control chart. Section "Performance Evaluation:" of this study is dedicated to the evaluation of the performance of the suggested control chart with the help of Monte Carlo simulation. An illustrative example is presented in Section "Iteration for Control Verification" to showcase the practical application of the suggested control chart. The final remarks are provided in Section "Application to Engine Oil Data".

## Design of proposed control chart

In this section, we discuss the proposed Max-EWMA control chart for joint monitoring of mean and variance with lognormal distribution. Let ${X}_{1},{X}_{2},{X}_{3},\dots , {X}_{i};i=1, 2, 3, 4,\dots n ,$ follow the lognormal distribution with respective location and scale parameters, that is *µ*_*x*_ and ${\sigma }_{x}$ with a probability density function

$$f\left(x\right)=\frac{1}{x{\sigma }_{x}\sqrt{2\pi }}exp\left(-\frac{{\left(lnx-{\mu }_{x}\right)}^{2}}{2{\sigma }_{x}^{2}}\right)$$

The CDF of lognormal distribution is

$$F\left(x\right)=\frac{1}{2}\left[1+erf\left(\frac{lnx-{\mu }_{x}}{{\sigma }_{x}\sqrt{2}}\right)\right]$$

To convert the mean and variance of a lognormal distribution to a normal distribution, one can use the properties of logarithms

1 $${\mu }_{y}=ln\left(\frac{{\mu }_{x}^{2}}{\sqrt{{\mu }_{x}^{2}+{\sigma }_{x}^{2}}}\right)$$

2 $${\sigma }_{y}^{2}=ln\left(1+\frac{{\sigma }_{x}^{2}}{{\mu }_{x}^{2}}\right)$$

For each data point *x* from the lognormal distribution, compute *Y* = ln(*x*). The resulting values of *Y* should follow a normal distribution with mean ${\mu }_{y}$ and standard deviation ${\sigma }_{y}$.

The current Shewhart control chart makes use of all the available information in the current sample. On the other hand, the EWMA control chart was designed to provide greater importance to the most recent subgroup, while assigning decreasing weights to previous observations in a geometric manner. The EWMA statistic is employed specifically to analyze shifts in the mean of a process. Roberts^23^ recommended EWMA statistic for the *j*th sample of size *n* for examining mean parameter as given in (3).

3 $${V}_{j}=\lambda {\overline{Y} }_{j}+(1-\lambda ){V}_{j-1}$$

Chen et al.^24^ introduced the concept of simultaneously monitoring both the mean shift and variance shift using a single control chart, which they referred to as the Max-EWMA control chart. Let *Y* represent a normally distributed random variable in the context of process production. The mean of *Y* is given by ${\mu }_{y}={\mu }_{0}+a{\sigma }_{0}$ and ${\sigma }_{y}^{2}={b}^{2}{\sigma }_{0}^{2}$ , where ${\mu }_{0}$ and ${\sigma }_{0}^{2}$ are respective parameters for the mean and variance when the process is in a stable state. The variables *a* and *b* represent shifts in mean and variance, respectively. In an in-control process, the values of *a* and *b* are 0 and 1, respectively. The utilization of Max-EWMA statistic has been found to exhibit enhanced efficiency in detecting minor shifts. The statistical properties of mean and variance for in-control methods, specifically for the *j*th sample, follow a normal distribution with a mean of zero and a variance of one.

4 $$U_{j} = \frac{{\overline{Y}_{j} - \mu_{0} }}{{\sqrt {\sigma_{0}^{2} /n} }},$$

5 $${V}_{j}={\varnothing }^{-1}\left[H\left\{\frac{\left(n-1\right){S}_{j}^{2}}{{\sigma }_{0}^{2}},(n-1)\right\}\right],$$

where *j* = 1, 2, 3,…,*n* is size of *j*th sample, the mean ${\overline{Y} }_{j}=\frac{\sum_{j=1}^{n}{Y}_{jk}}{n}$ of *j*th sample, and variance ${S}_{j}^{2}=\frac{{\sum }_{j=1}^{n}{\left({Y}_{jk}-{\overline{Y} }_{j}\right)}^{2}}{n-1}$ of *j*th sample, The inverse function of the usual normal distribution is denoted as ${\varnothing }^{-1}$. On the other hand, the random variable $H(\xi ,v)$ follows a chi-squared distribution with *n−1* degrees of freedom.

The two EWMA statistics can be derived by utilizing Eqs. (4) and (5).

6 $${P}_{j}=\lambda {U}_{j}+\left(1-\lambda \right){U}_{j-1},$$

7 $${Q}_{j}=\lambda {V}_{j}+(1-\lambda ){V}_{j-1},$$

In the first sample, the values of ${P}_{0}$ and ${Q}_{0}$ are both set to zero. The smoothing constant, denoted as λ, is defined such that it falls within the range of 0–1, inclusive.

In the suggested statistical framework, the sum of all weights is equal to one. The variables ${U}_{j-1}$ and ${V}_{j-1}$ represent the previous values of the respective quantities. In order to streamline the analysis of mean and variance, we propose employing a single maximum absolute statistic for their joint monitoring of mean and variance, as opposed to analyzing these statistics individually.

8 $${T}_{j}=\text{ Max }\left(\left|{P}_{j}\right|,|{Q}_{j}|\right)$$

A single upper control limit (*UCL*) is sufficient for the representation of Max-EWMA statistic to regulate the production process. The *UCL* in Eq. (9) draws inspiration from the research conducted by Chen et al.^24^. For further insights into the specific values employed in Eq. (9), readers are encouraged to refer to the work of Chen et al.^24^.

9 $$UCL=(1.128379+0.602810\times L)\sqrt{Var\left({P}_{j}\right)}$$

The control constant indicated as *L*, is determined for achieving the desired average run length (ARL_0_) in an in-control process. Additionally, the variance is also included in this calculation.

10 $$Var\left({P}_{j}\right)=Var\left({Q}_{j}\right)=\sqrt{\frac{\lambda }{(2-\lambda )}}$$

## Performance evaluation

The evaluation of the control chart is typically conducted by the analysis of its run length attributes, ARLs and SDRLs. When the process is under control, it is expected that a control chart would possess a large value of ARL, indicating that control chart would infrequently signal any deviations from the established control limits. In contrast, in instances where the process is not under control, a control chart should possess a small ARL, indicating its ability to promptly detect signals indicating an out-of-control state. In the context of control charts, it is generally regarded that the control chart exhibiting a reduced ARL when subjected to a particular shift parameter is deemed to be superior in terms of performance. When the process is operating in a state of control, it is necessary to implement measures to minimize the occurrence of frequent false alarms. To promptly identify any shifts in the out-of-control process, it is imperative that the ARL be minimized. The ARL and SDRL at any shift are denoted by ARL_1_ and SDRL_1._ A lower value of SDRL serves as an indicator of improved performance of a control chart. Monte Carlo simulations are employed to calculate the ARLs with each run consisting of 30,000 repeats. During the duration of the analysis, it is necessary for the calculated value of the plotting statistic ${T}_{j}$ to fall under the *UCL*. The following steps are considered to compute the run length profile:

### Initialization

Select the parameters of the lognormal distribution *L*($\alpha$_0_, $\gamma$_0_) and transform them into the parameters of normal distribution by using the Eqs. (1) and (2).

### Sample generation

Obtain the sample of size *n* by using the transformed parameters of standard normal distribution and calculate the statistic of the proposed control chart by using Eq. (8).

### Set up the *UCL*

Set up the *UCL* by selecting the initial value of the control constant (*L*) as described in Eq. (9) and compare the plotting statistic with *UCL*.

### Iteration for control verification

Continue iterating the last three steps of the procedure until it's verified to be within control. If the process is identified as out of control, note down the count of in-control occurrences as the run length.

### ARL_0_ calculation

Compute ARL_0_ by repeating the above steps (1–4) for 30,000 times. Should the target ARL_0_ not be achieved, redo the preceding steps by using a different value for *L*. The selected value of *L* for a specific ARL_0_ will be used in the next step.

6. ARL_1_ calculation

Select the shifted parameters of the lognormal distribution *L*($\alpha$, $\gamma$). Repeat the steps 1 to 4 and compute the value of ARL_1_ by repeating the process 30,000 times.

The shift occurs when the value of the plotted statistic falls outside the *UCL*. The impact of variations in lognormal parameters on the process mean and process standard deviation is taken into consideration. In this context, the shape parameters undergo variations at the values of 0.5, 1, 1.25, 1.5, 1.75, 1.9, 2, 2.16, 2.25, 2.5, 2.75, 3.00, 4.00 and 5.00. While the scale parameters experience changes at the values of 0.25, 0.5, 0.75, 1, 1.25, 1.5, 1.649, 1.75, 2, 2.25, 2.5, 2.75, 3.00, 4.00, and 5.00. The process at point *L*(1.65, 2.16) is determined to be in-control, with the associated normal process following a distribution of *N*(0, 1). The value of the in-control ARL_0_ is set to 370. With two different settings of smoothing parameter $\lambda$ = 0.3 and 0.2. The corresponding values of *L* are 3.345 and 3.252, respectively. The ARLs and SDRLs for the proposed Max-EWMA control chart, considering different changes in shape and scale parameters, are presented in Tables 1 and 2, respectively. The bold values of ARLs and SDRLs are presented in Tables 1 and 2 for the fixed value of the scale parameter and various values of the shape parameter. The parametric settings to calculate the ARLs and SDRLs are presented in Table 3.

**Table 1:** *ARLs* and *SDRLs* of Max-EWMA chart in lognormal parameters *L*($\alpha$_0_ = 1.649, $\gamma$_0_ = 2.16) using *L* = 3.345 for $\lambda \hspace{0.17em}$= 0.3 for *ARL*_*0*_ = 370 with sample size *n* = 3, 5 and 10.

		0.5	1	1.25	1.5	1.75	1.9	2.16
		ARL (SDRL)	ARL (SDRL)	ARL (SDRL)	ARL (SDRL)	ARL (SDRL)	ARL (SDRL)	ARL (SDRL)
Shape parameter$\gamma$								
$\alpha$	(µ, σ)	(− 2.191, 1.6094)	(− 2.8029, 2.8332)	(− 3.0153, 3.2581)	(− 3.1918, 3.6109)	(− 3.3423, 3.912)	(− 3.423, 4.0735)	(− 3.5499, 4.3272)
0.25	3	1.79	1.40	1.30	1.25	1.21	1.20	1.17
		(0.72)	(0.60)	(0.54)	(0.50)	(0.46)	(0.44)	(0.41)
	5	1.39	1.14	1.10	1.07	1.05	1.05	1.04
		(0.52)	(0.36)	(0.30)	(0.26)	(0.23)	(0.22)	(0.19)
	10	1.05	1.01	1.00	1.00	1.00	1.00	1.00
		(0.23)	(0.10)	(0.07)	(0.05)	(0.04)	(0.03)	(0.03)
	(µ, σ)	(− 1.0397, 0.6931)	(− 1.4979, 1.6094)	(− 1.6836, 1.981)	(− 1.8444, 2.3026)	(− 1.9851, 2.584)	(− 2.0616, 2.737)	(− 2.183, 2.9797)
0.5	3	4.40	2.62	2.20	1.91	1.73	1.66	1.54
		(1.57)	(1.22)	(1.06)	(0.92)	(0.83)	(0.79)	(0.72)
	5	2.97	1.98	1.68	1.48	1.35	1.30	1.23
		(0.83)	(0.80)	(0.71)	(0.62)	(0.54)	(0.50)	(0.45)
	10	2.03	1.40	1.22	1.12	1.06	1.04	1.03
		(0.36)	(0.53)	(0.43)	(0.32)	(0.25)	(0.21)	(0.16)
	(µ, σ)	(− 0.4715, 0.3677)	(− 0.7985, 1.0217)	(− 0.9523, 1.3291)	(− 1.0924, 1.6094)	(− 1.2193, 1.8632)	(− 1.2896, 2.0039)	(− 1.4029, 2.2304)
0.75	3	7.90	6.86	4.68	3.57	2.88	2.64	2.30
		(3.95)	(4.00)	(2.62)	(1.90)	(1.51)	(1.36)	(1.18)
	5	3.59	4.43	3.34	2.61	2.16	1.98	1.73
		(1.00)	(2.09)	(1.56)	(1.18)	(0.96)	(0.87)	(0.76)
	10	2.08	2.75	2.23	1.80	1.52	1.40	1.24
		(0.34)	(0.97)	(0.83)	(0.69)	(0.59)	(0.54)	(0.44)
	(µ, σ)	(− 0.1116, 0.2231)	(− 0.3466, 0.6931)	(− 0.4705, 0.941)	(− 0.5893, 1.1787)	(− 0.7009, 1.4018)	(− 0.7641, 1.5282)	(− 0.8677, 1.7353)
1	3	4.12	65.90	23.54	10.61	6.45	5.26	3.99
		(1.16)	(61.09)	(19.40)	(7.63)	(4.15)	(3.27)	(2.31)
	5	2.35	19.98	12.59	6.83	4.46	3.67	2.82
		(0.49)	(15.16)	(8.83)	(4.20)	(2.44)	(1.88)	(1.37)
	10	1.67	6.71	6.09	4.01	2.86	2.42	1.92
		(0.47)	(3.24)	(3.25)	(1.90)	(1.21)	(0.98)	(0.75)
	(µ,σ)	(0.1489,0.1484)	(− 0.0242,0.4947)	(− 0.1234,0.6931)	(− 0.2229,0.892)	(− 0.3195,1.0852)	(− 0.3754,1.1971)	(− 0.4687,1.3836)
1.25	3	3.13	17.47	88.19	164.09	38.02	20.30	9.89
		(0.65)	(12.71)	(83.53)	(161.42)	(34.77)	(16.99)	(7.32)
	5	2.00	6.04	25.07	84.64	23.11	12.81	6.46
		(0.14)	(2.67)	(20.53)	(80.31)	(19.56)	(9.85)	(4.06)
	10	1.09	2.89	7.69	31.94	11.56	7.05	3.90
		(0.28)	(0.77)	(4.21)	(27.33)	(8.41)	(4.35)	(1.92)
	(µ, σ)	(0.2358, 0.1285)	(0.0814, 0.4374)	(− 0.0095, 0.6191)	(− 0.1019, 0.804)	(− 0.1929, 0.986)	(− 0.246, 1.0922)	(− 0.3352, 1.2705)
1.35	3	2.93	11.81	46.28	229.43	143.01	55.49	18.10
		(0.56)	(7.41)	(41.71)	(225.71)	(138.28)	(52.14)	(15.08)
	5	1.98	4.65	13.13	87.84	91.81	35.35	11.41
		(0.14)	(1.66)	(8.87)	(83.47)	(88.19)	(31.64)	(8.54)
	10	1.02	2.44	4.82	24.58	43.26	18.12	6.37
		(0.13)	(0.57)	(1.95)	(20.28)	(39.03)	(14.58)	(3.84)
	(µ, σ)	(0.456, 0.088)	(0.3434, 0.3133)	(0.2729, 0.4541)	(0.1984, 0.6031)	(0.1227, 0.7546)	(0.0775, 0.845)	(0, 1)
1.649	3	2.53	6.04	13.16	40.48	151.18	315.33	370.95
		(0.51)	(2.46)	(8.71)	(35.34)	(145.30)	(311.12)	(370.73)
	5	1.93	3.03	4.98	11.60	48.20	145.05	371.04
		(0.25)	(0.72)	(1.88)	(7.41)	(43.15)	(140.30)	(366.60)
	10	1.00	1.96	2.56	4.46	13.38	46.34	371.20
		(0.00)	(0.24)	(0.62)	(1.70)	(9.30)	(41.79)	(369.84)
	(µ, σ)	(0.5204, 0.0785)	(0.4183, 0.2826)	(0.3535, 0.4122)	(0.2842, 0.5508)	(0.213, 0.6931)	(0.1702, 0.7788)	(0.0965, 0.9263)
1.75	3	2.41	5.27	10.20	26.55	85.18	172.16	373.56
		(0.49)	(1.89)	(6.00)	(21.55)	(79.90)	(166.34)	(372.50)
	5	1.90	2.77	4.21	8.14	24.69	60.60	265.12
		(0.30)	(0.62)	(1.38)	(4.40)	(20.10)	(55.76)	(262.49)
	10	1.00	1.90	2.29	3.53	7.62	17.10	137.13
		(0.00)	(0.31)	(0.49)	(1.12)	(4.17)	(12.91)	(132.11)
	(µ, σ)	(0.6628, 0.0606)	(0.5816, 0.2231)	(0.5283, 0.3298)	(0.47, 0.4463)	(0.4089, 0.5685)	(0.3716, 0.6432)	(0.3063, 0.7736)
2	3	2.18	4.12	6.54	12.43	28.32	47.72	98.41
		(0.39)	(1.16)	(2.81)	(7.92)	(23.09)	(42.35)	(93.79)
	5	1.78	2.35	3.18	4.80	8.78	14.13	35.77
		(0.42)	(0.49)	(0.79)	(1.75)	(4.79)	(9.49)	(30.97)
	10	1.00	1.67	1.98	2.50	3.72	5.15	11.21
		(0.00)	(0.47)	(0.24)	(0.59)	(1.18)	(2.05)	(7.03)
	(µ, σ)	(0.7868, 0.0482)	(0.7208, 0.1803)	(0.6764, 0.269)	(0.6271, 0.3677)	(0.5744, 0.4731)	(0.5418, 0.5383)	(0.4841, 0.6537)
2.25	3	2.05	3.50	4.95	7.77	13.03	18.38	30.62
		(0.22)	(0.82)	(1.67)	(3.71)	(8.25)	(13.05)	(25.13)
	5	1.59	2.09	2.68	3.57	5.12	6.56	10.75
		(0.49)	(0.31)	(0.59)	(0.97)	(1.83)	(2.80)	(6.30)
	10	1.00	1.33	1.87	2.08	2.66	3.19	4.56
		(0.00)	(0.47)	(0.34)	(0.32)	(0.63)	(0.84)	(1.58)
	(µ, σ)	(0.8967, 0.0392)	(0.8421, 0.1484)	(0.8047, 0.2231)	(0.7625, 0.3075)	(0.7169, 0.3988)	(0.6883, 0.4559)	(0.6373, 0.5581)
2.5	3	2.00	3.13	4.11	5.63	7.78	9.50	13.29
		(0.10)	(0.64)	(1.11)	(1.93)	(3.37)	(4.74)	(8.19)
	5	1.37	2.00	2.34	2.96	3.79	4.42	5.81
		(0.48)	(0.15)	(0.49)	(0.66)	(0.99)	(1.28)	(2.17)
	10	1.00	1.08	1.67	1.94	2.18	2.47	3.06
		(0.00)	(0.27)	(0.47)	(0.25)	(0.41)	(0.54)	(0.73)
	(µ, σ)	(0.9953, 0.0325)	(0.9495, 0.1242)	(0.9177, 0.1878)	(0.8814, 0.2605)	(0.8416, 0.34)	(0.8165, 0.3903)	(0.7711, 0.4809)
2.75	3	2.00	2.88	3.59	4.50	5.57	6.32	7.83
		(0.08)	(0.55)	(0.83)	(1.16)	(1.67)	(2.15)	(3.33)
	5	1.19	1.98	2.13	2.59	3.12	3.47	4.15
		(0.39)	(0.14)	(0.35)	(0.54)	(0.66)	(0.77)	(1.10)
	10	1.00	1.01	1.40	1.84	1.98	2.08	2.42
		(0.00)	(0.10)	(0.49)	(0.37)	(0.21)	(0.31)	(0.52)
	(µ, σ)	(1.0849, 0.0274)	(1.0459, 0.1054)	(1.0186, 0.1601)	(0.987, 0.2231)	(0.9522, 0.2929)	(0.93, 0.3373)	(0.8896, 0.418)
3	3	1.99	2.70	3.23	3.85	4.47	4.85	5.59
		(0.09)	(0.51)	(0.65)	(0.80)	(1.00)	(1.19)	(1.69)
	5	1.07	1.96	2.03	2.34	2.74	2.95	3.34
		(0.25)	(0.19)	(0.20)	(0.48)	(0.51)	(0.54)	(0.68)
	10	1.00	1.00	1.16	1.67	1.92	1.97	2.06
		(0.00)	(0.02)	(0.37)	(0.47)	(0.27)	(0.18)	(0.28)
	(µ, σ)	(1.3785, 0.0155)	(1.356, 0.0606)	(1.3397, 0.0932)	(1.3205, 0.1316)	(1.2987, 0.1751)	(1.2846, 0.2035)	(1.2582, 0.2561)
4	3	1.98	2.17	2.57	2.81	2.92	2.97	3.07
		(0.15)	(0.38)	(0.50)	(0.39)	(0.29)	(0.28)	(0.37)
	5	1.00	1.78	1.94	1.98	2.00	2.00	2.06
		(0.01)	(0.42)	(0.23)	(0.13)	(0.07)	(0.09)	(0.24)
	10	1.00	1.00	1.00	1.02	1.27	1.50	1.78
		(0.00)	(0.00)	(0.01)	(0.15)	(0.44)	(0.50)	(0.41)
	(µ, σ)	(1.6045, 0.01)	(1.5898, 0.0392)	(1.5791, 0.0606)	(1.5663, 0.0862)	(1.5517, 0.1156)	(1.542, 0.1349)	(1.5238, 0.1713)
5	3	1.95	2.00	2.00	2.01	2.09	2.17	2.32
		(0.22)	(0.07)	(0.04)	(0.11)	(0.28)	(0.38)	(0.47)
	5	1.00	1.37	1.78	1.93	1.97	1.99	1.99
		(0.00)	(0.48)	(0.42)	(0.26)	(0.16)	(0.12)	(0.08)
	10	1.00	1.00	1.00	1.00	1.00	1.00	1.00
		(0.00)	(0.00)	(0.00)	(0.00)	(0.00)	(0.00)	(0.04)

		2.25	2.5	2.75	3	3.5	4	5
		ARL (SDRL)	ARL (SDRL)	ARL (SDRL)	ARL (SDRL)	ARL (SDRL)	ARL (SDRL)	ARL (SDRL)
Shape parameter$\gamma$								
	(µ, σ)	(− 3.5897, 4.4067)	(− 3.6939, 4.6151)	(− 3.7883, 4.804)	(− 3.8747, 4.9767)	(− 4.0279, 5.2832)	(− 4.1608, 5.5491)	(− 4.3833, 5.994)
0.25	3	1.16	1.15	1.14	1.13	1.11	1.10	1.08
		(0.40)	(0.39)	(0.37)	(0.36)	(0.33)	(0.31)	(0.29)
	5	1.03	1.03	1.03	1.02	1.02	1.01	1.01
		(0.18)	(0.17)	(0.16)	(0.15)	(0.14)	(0.12)	(0.11)
	10	1.00	1.00	1.00	1.00	1.00	1.00	1.00
		(0.03)	(0.01)	(0.02)	(0.01)	(0.01)	(0.01)	(0.01)
	(µ,σ)	(− 2.2213,3.0564)	(− 2.3222,3.2581)	(− 2.4142,3.442)	(− 2.4986,3.6109)	(− 2.6492,3.912)	(− 2.7803,4.1744)	(− 3.0007,4.6151)
0.5	3	1.51	1.45	1.39	1.36	1.30	1.25	1.20
		(0.70)	(0.66)	(0.61)	(0.59)	(0.54)	(0.49)	(0.44)
	5	1.22	1.17	1.14	1.12	1.09	1.07	1.05
		(0.44)	(0.40)	(0.36)	(0.34)	(0.29)	(0.26)	(0.21)
	10	1.02	1.01	1.01	1.01	1.00	1.00	1.00
		(0.15)	(0.12)	(0.10)	(0.08)	(0.06)	(0.05)	(0.04)
	(µ,σ)	(− 1.439,2.3026)	(− 1.5347,2.4941)	(− 1.6228,2.6703)	(− 1.7043,2.8332)	(− 1.8506,3.1258)	(− 1.9789,3.3825)	(− 2.1959,3.8165)
0.75	3	2.21	2.01	1.87	1.74	1.60	1.49	1.37
		(1.12)	(1.01)	(0.94)	(0.86)	(0.77)	(0.69)	(0.60)
	5	1.66	1.53	1.43	1.36	1.26	1.19	1.12
		(0.73)	(0.66)	(0.60)	(0.55)	(0.48)	(0.42)	(0.34)
	10	1.21	1.14	1.09	1.06	1.03	1.02	1.01
		(0.42)	(0.35)	(0.29)	(0.25)	(0.18)	(0.14)	(0.09)
	(µ,σ)	(− 0.9011,1.8021)	(− 0.9905,1.981)	(− 1.0737,2.1474)	(− 1.1513,2.3026)	(− 1.292,2.584)	(− 1.4166,2.8332)	(− 1.629,3.2581)
1	3	3.69	3.10	2.71	2.43	2.07	1.86	1.61
		(2.11)	(1.73)	(1.48)	(1.29)	(1.07)	(0.95)	(0.79)
	5	2.64	2.26	2.00	1.81	1.57	1.42	1.26
		(1.25)	(1.04)	(0.92)	(0.82)	(0.68)	(0.59)	(0.48)
	10	1.80	1.56	1.40	1.28	1.15	1.09	1.03
		(0.70)	(0.61)	(0.54)	(0.47)	(0.36)	(0.28)	(0.18)
	(µ,σ)	(− 0.4991,1.4446)	(− 0.5816,1.6094)	(− 0.6592,1.7647)	(− 0.7324,1.911)	(− 0.8665,2.1793)	(− 0.9866,2.4195)	(− 1.1935,2.8332)
1.25	3	8.30	5.79	4.52	3.73	2.89	2.43	1.95
		(5.93)	(3.78)	(2.78)	(2.21)	(1.63)	(1.31)	(1.01)
	5	5.49	3.93	3.12	2.64	2.08	1.78	1.47
		(3.28)	(2.13)	(1.57)	(1.28)	(0.97)	(0.80)	(0.63)
	10	3.39	2.53	2.07	1.78	1.45	1.26	1.10
		(1.59)	(1.06)	(0.84)	(0.70)	(0.57)	(0.46)	(0.31)
	(µ,σ)	(− 0.3645,1.3291)	(− 0.444,1.4883)	(− 0.5193,1.6389)	(− 0.5906,1.7814)	(− 0.7219,2.044)	(− 0.84,2.2803)	(− 1.0444,2.689)
1.35	3	13.99	8.28	5.88	4.63	3.35	2.74	2.11
		(11.10)	(5.93)	(3.91)	(2.92)	(1.93)	(1.51)	(1.12)
	5	8.87	5.44	3.94	3.19	2.38	1.98	1.57
		(6.21)	(3.30)	(2.14)	(1.63)	(1.13)	(0.92)	(0.69)
	10	5.07	3.30	2.51	2.09	1.61	1.38	1.15
		(2.80)	(1.54)	(1.07)	(0.85)	(0.64)	(0.54)	(0.36)
	(µ,σ)	(− 0.0259,1.0517)	(− 0.0969,1.1937)	(− 0.1652,1.3303)	(− 0.2306,1.4612)	(− 0.353,1.706)	(− 0.4648,1.9295)	(− 0.6611,2.3221)
1.649	3	222.63	51.99	20.45	11.60	6.01	4.18	2.81
		(218.01)	(48.97)	(17.89)	(9.05)	(4.08)	(2.57)	(1.59)
	5	199.00	32.80	12.24	7.02	3.91	2.86	2.01
		(195.54)	(29.43)	(9.41)	(4.64)	(2.17)	(1.46)	(0.94)
	10	155.84	16.66	6.44	4.00	2.46	1.87	1.37
		(152.34)	(13.33)	(4.00)	(2.07)	(1.06)	(0.76)	(0.53)
	(µ,σ)	(0.0718,0.9757)	(0.0036,1.1121)	(− 0.0624,1.244)	(− 0.1258,1.3709)	(− 0.2451,1.6094)	(− 0.3546,1.8285)	(− 0.548,2.2152)
1.75	3	361.84	120.82	38.11	17.73	7.71	4.94	3.11
		(357.08)	(118.14)	(34.81)	(14.90)	(5.60)	(3.19)	(1.79)
	5	314.82	91.56	22.91	10.57	4.89	3.30	2.20
		(309.74)	(87.88)	(19.98)	(7.95)	(2.97)	(1.74)	(1.05)
	10	230.73	53.01	11.37	5.61	2.93	2.12	1.49
		(225.92)	(49.61)	(8.47)	(3.31)	(1.35)	(0.88)	(0.59)
	(µ,σ)	(0.2842,0.8179)	(0.2227,0.941)	(0.1624,1.0615)	(0.1038,1.1787)	(− 0.0078,1.4018)	(− 0.1116,1.6094)	(− 0.2974,1.981)
2	3	115.80	138.84	110.96	57.48	16.53	8.25	4.19
		(110.69)	(134.53)	(106.87)	(54.69)	(13.93)	(6.13)	(2.62)
	5	47.52	78.86	77.98	36.47	9.67	5.09	2.80
		(42.48)	(75.08)	(74.47)	(33.00)	(7.15)	(3.13)	(1.43)
	10	15.40	32.93	43.09	18.89	5.13	3.00	1.83
		(11.15)	(28.75)	(39.67)	(15.72)	(2.94)	(1.38)	(0.76)
	(µ,σ)	(0.4644,0.6931)	(0.4089,0.804)	(0.354,0.9138)	(0.3001,1.0217)	(0.1961,1.2296)	(0.0981,1.4256)	(− 0.0798,1.7814)
2.25	3	35.03	46.05	52.65	54.28	32.31	14.45	5.84
		(29.46)	(41.26)	(47.55)	(50.55)	(28.99)	(11.76)	(3.91)
	5	12.67	19.54	26.33	31.67	19.98	8.59	3.72
		(8.11)	(15.17)	(21.91)	(27.77)	(16.69)	(6.10)	(2.08)
	10	5.19	7.65	10.95	14.60	10.34	4.66	2.30
		(1.99)	(4.10)	(7.44)	(11.02)	(7.47)	(2.58)	(0.99)
	(µ,σ)	(0.6196,0.5933)	(0.5697,0.6931)	(0.5198,0.793)	(0.4703,0.892)	(0.3737,1.0852)	(0.2814,1.2698)	(0.1116,1.6094)
2.5	3	14.71	18.67	22.58	25.75	28.59	20.86	8.22
		(9.58)	(13.74)	(17.89)	(21.31)	(25.03)	(17.75)	(6.05)
	5	6.38	8.32	10.58	13.18	17.20	13.01	5.11
		(2.59)	(4.44)	(6.63)	(9.25)	(13.88)	(10.15)	(3.14)
	10	3.31	4.05	4.98	6.09	8.70	7.12	3.00
		(0.85)	(1.31)	(2.07)	(3.13)	(5.72)	(4.55)	(1.40)
	(µ, σ)	(0.7554, 0.5125)	(0.7104, 0.6024)	(0.665, 0.6931)	(0.6196, 0.7839)	(0.53, 0.9631)	(0.4434, 1.1365)	(0.2816, 1.46)
2.75	3	8.33	10.06	11.93	13.60	17.01	18.44	10.91
		(3.85)	(5.57)	(7.42)	(9.42)	(13.19)	(15.07)	(8.50)
	5	4.39	5.18	6.11	7.16	9.48	11.41	6.78
		(1.26)	(1.83)	(2.68)	(3.74)	(6.16)	(8.24)	(4.47)
	10	2.56	2.91	3.32	3.79	4.94	6.17	3.91
		(0.56)	(0.70)	(0.96)	(1.34)	(2.39)	(3.59)	(1.99)
	(µ, σ)	(0.8755, 0.4463)	(0.8349, 0.5274)	(0.7937, 0.6099)	(0.752, 0.6931)	(0.669, 0.8591)	(0.5878, 1.0217)	(0.434, 1.3291)
3	3	5.84	6.72	7.62	8.57	10.65	12.64	12.05
		(1.90)	(2.66)	(3.60)	(4.60)	(6.93)	(9.21)	(9.32)
	5	3.48	3.91	4.37	4.87	6.10	7.54	7.73
		(0.77)	(1.03)	(1.40)	(1.85)	(3.07)	(4.62)	(5.22)
	10	2.12	2.36	2.60	2.84	3.42	4.16	4.55
		(0.35)	(0.50)	(0.61)	(0.76)	(1.23)	(1.90)	(2.37)
	(µ, σ)	(1.2488, 0.2749)	(1.2214, 0.3298)	(1.1928, 0.3871)	(1.1632, 0.4463)	(1.102, 0.5685)	(1.0397, 0.6931)	(0.9158, 0.941)
4	3	3.12	3.25	3.40	3.57	3.94	4.39	5.47
		(0.41)	(0.53)	(0.64)	(0.79)	(1.13)	(1.59)	(2.72)
	5	2.10	2.23	2.36	2.48	2.71	2.95	3.61
		(0.30)	(0.42)	(0.48)	(0.52)	(0.63)	(0.82)	(1.41)
	10	1.84	1.92	1.93	1.94	1.96	2.03	2.34
		(0.37)	(0.28)	(0.25)	(0.23)	(0.26)	(0.37)	(0.68)
	(µ, σ)	(1.5172, 0.1844)	(1.4979, 0.2231)	(1.4773, 0.2643)	(1.4557, 0.3075)	(1.41, 0.3988)	(1.3621, 0.4947)	(1.2629, 0.6931)
5	3	2.36	2.47	2.56	2.62	2.73	2.87	3.22
		(0.48)	(0.50)	(0.50)	(0.49)	(0.52)	(0.62)	(0.95)
	5	2.00	2.00	2.00	2.00	2.02	2.10	2.34
		(0.07)	(0.04)	(0.03)	(0.04)	(0.15)	(0.31)	(0.54)
	10	1.01	1.05	1.13	1.24	1.44	1.57	1.73
		(0.07)	(0.21)	(0.34)	(0.43)	(0.50)	(0.50)	(0.45)

**Table 2:** *ARLs* and *SDRLs* of Max-EWMA chart in lognormal parameters *L* ($\alpha$_0_ = 1.649, $\gamma$_0_ = 2.16) using *L* = 3.252 for $\lambda$ =0.2 for *ARL*_*0*_ = 370 with sample size *n* = 3,5,10.

		0.5	1	1.25	1.5	1.75	1.9	2.16
		ARL (SDRL)	ARL (SDRL)	ARL (SDRL)	ARL (SDRL)	ARL (SDRL)	ARL (SDRL)	ARL (SDRL)
Shape parameter$\gamma$								
$\alpha$	(µ, σ)	(− 2.191, 1.6094)	(− 2.8029, 2.8332)	(− 3.0153, 3.2581)	(− 3.1918, 3.6109)	(− 3.3423, 3.912)	(− 3.5499, 4.3272)	(− 3.5499, 4.3272)
0.25	3	2.03	1.55	1.44	1.37	1.31	1.28	1.25
		(0.70)	(0.66)	(0.62)	(0.57)	(0.52)	(0.50)	(0.48)
	5	1.59	1.24	1.17	1.13	1.10	1.09	1.07
		(0.56)	(0.44)	(0.38)	(0.34)	(0.30)	(0.28)	(0.26)
	10	1.13	1.03	1.01	1.01	1.00	1.00	1.00
		(0.34)	(0.16)	(0.11)	(0.08)	(0.06)	(0.06)	(0.05)
	(µ, σ)	(− 1.0397, 0.6931)	(− 1.4979, 1.6094)	(− 1.6836, 1.981)	(− 1.8444, 2.3026)	(− 1.9851, 2.584)	(− 2.0616, 2.737)	(− 2.183, 2.9797)
0.5	3	4.46	2.90	2.46	2.18	1.96	1.87	1.73
		(1.27)	(1.19)	(1.07)	(0.97)	(0.88)	(0.85)	(0.78)
	5	3.20	2.23	1.91	1.69	1.53	1.45	1.36
		(0.75)	(0.79)	(0.72)	(0.66)	(0.61)	(0.57)	(0.53)
	10	2.19	1.62	1.39	1.23	1.13	1.10	1.06
		(0.41)	(0.56)	(0.51)	(0.42)	(0.34)	(0.30)	(0.24)
	(µ, σ)	(− 0.4715, 0.3677)	(− 0.7985, 1.0217)	(− 0.9523, 1.3291)	(− 1.0924, 1.6094)	(− 1.2193, 1.8632)	(− 1.2896, 2.0039)	(− 1.4029, 2.2304)
0.75	3	6.83	6.57	4.92	3.89	3.24	2.95	2.60
		(2.36)	(3.15)	(2.40)	(1.84)	(1.53)	(1.38)	(1.21)
	5	3.76	4.59	3.59	2.90	2.44	2.24	1.97
		(0.84)	(1.82)	(1.45)	(1.16)	(0.95)	(0.88)	(0.78)
	10	2.26	2.98	2.48	2.06	1.75	1.61	1.42
		(0.44)	(0.91)	(0.80)	(0.67)	(0.60)	(0.58)	(0.52)
	(µ, σ)	(− 0.1116, 0.2231)	(− 0.3466, 0.6931)	(− 0.4705, 0.941)	(− 0.5893, 1.1787)	(− 0.7009, 1.4018)	(− 0.7641, 1.5282)	(− 0.8677, 1.7353)
1	3	4.20	34.82	17.95	10.01	6.70	5.58	4.36
		(0.91)	(27.32)	(12.56)	(6.24)	(3.81)	(3.05)	(2.26)
	5	2.65	13.88	10.59	6.74	4.72	3.98	3.16
		(0.50)	(7.83)	(6.10)	(3.52)	(2.23)	(1.79)	(1.35)
	10	1.97	6.28	5.90	4.25	3.15	2.71	2.20
		(0.17)	(2.20)	(2.52)	(1.74)	(1.17)	(0.95)	(0.74)
	(µ, σ)	(0.1489, 0.1484)	(− 0.0242, 0.4947)	(− 0.1234, 0.6931)	(− 0.2229, 0.892)	(− 0.3195, 1.0852)	(− 0.3754, 1.1971)	(− 0.4687, 1.3836)
1.25	3	3.36	12.26	51.20	106.88	31.32	18.28	9.90
		(0.58)	(6.60)	(44.61)	(99.68)	(26.44)	(13.85)	(6.53)
	5	2.11	5.66	17.27	54.76	19.41	11.96	6.67
		(0.31)	(1.85)	(11.35)	(47.55)	(14.53)	(7.96)	(3.65)
	10	1.68	3.13	6.98	22.41	10.46	7.01	4.21
		(0.47)	(0.70)	(2.92)	(16.22)	(6.40)	(3.69)	(1.80)
	(µ, σ)	(0.2358, 0.1285)	(0.0814, 0.4374)	(− 0.0095, 0.6191)	(− 0.1019, 0.804)	(− 0.1929, 0.986)	(− 0.246, 1.0922)	(− 0.3352, 1.2705)
1.35	3	3.16	9.18	27.31	146.36	109.94	46.79	16.99
		(0.51)	(4.08)	(20.53)	(139.36)	(103.11)	(41.40)	(12.90)
	5	2.02	4.61	10.24	53.97	67.29	29.41	10.98
		(0.16)	(1.26)	(5.19)	(47.29)	(61.10)	(24.33)	(7.22)
	10	1.46	2.70	4.80	17.66	32.08	15.55	6.49
		(0.50)	(0.57)	(1.54)	(11.91)	(26.45)	(10.96)	(3.32)
	(µ, σ)	(0.456, 0.088)	(0.3434, 0.3133)	(0.2729, 0.4541)	(0.1984, 0.6031)	(0.1227, 0.7546)	(0.0775, 0.845)	(0,1)
1.649	3	2.83	5.62	9.90	24.23	89.97	214.92	370.36
		(0.41)	(1.62)	(4.68)	(17.61)	(82.77)	(208.66)	(363.47)
	5	2.00	3.26	4.89	9.31	30.38	92.93	370.95
		(0.05)	(0.63)	(1.41)	(4.46)	(23.74)	(86.02)	(365.67)
	10	1.03	2.04	2.82	4.50	10.81	30.96	370.33
		(0.17)	(0.22)	(0.60)	(1.36)	(5.90)	(24.50)	(365.89)
	(µ, σ)	(0.5204, 0.0785)	(0.4183, 0.2826)	(0.3535, 0.4122)	(0.2842, 0.5508)	(0.213, 0.6931)	(0.1702, 0.7788)	(0.0965, 0.9263)
1.75	3	2.75	5.06	8.22	16.89	48.95	103.18	299.61
		(0.44)	(1.32)	(3.33)	(10.72)	(41.51)	(95.67)	(293.59)
	5	2.00	3.04	4.28	7.15	16.86	37.31	195.89
		(0.05)	(0.55)	(1.09)	(2.83)	(10.87)	(30.31)	(190.48)
	10	1.01	2.00	2.54	3.72	6.90	13.00	96.08
		(0.07)	(0.12)	(0.54)	(0.96)	(2.85)	(7.63)	(90.19)
	(µ, σ)	(0.6628, 0.0606)	(0.5816, 0.2231)	(0.5283, 0.3298)	(0.47, 0.4463)	(0.4089, 0.5685)	(0.3716, 0.6432)	(0.3063, 0.7736)
2	3	2.56	4.20	5.94	9.42	17.24	26.16	53.73
		(0.50)	(0.90)	(1.81)	(4.13)	(10.58)	(18.84)	(46.15)
	5	1.99	2.66	3.40	4.76	7.48	10.59	22.20
		(0.09)	(0.50)	(0.69)	(1.32)	(2.89)	(5.10)	(15.43)
	10	1.00	1.97	2.09	2.76	3.90	5.09	9.31
		(0.01)	(0.18)	(0.29)	(0.58)	(1.01)	(1.57)	(4.33)
	(µ, σ)	(0.7868, 0.0482)	(0.7208, 0.1803)	(0.6764, 0.269)	(0.6271, 0.3677)	(0.5744, 0.4731)	(0.5418, 0.5383)	(0.4841, 0.6537)
2.25	3	2.33	3.68	4.83	6.64	9.41	11.78	17.59
		(0.47)	(0.70)	(1.18)	(2.05)	(3.75)	(5.48)	(10.67)
	5	1.98	2.34	2.94	3.74	5.01	6.06	8.63
		(0.14)	(0.47)	(0.52)	(0.82)	(1.33)	(1.84)	(3.50)
	10	1.00	1.88	1.99	2.27	2.93	3.44	4.64
		(0.00)	(0.33)	(0.11)	(0.44)	(0.60)	(0.75)	(1.26)
	(µ, σ)	(0.8967, 0.0392)	(0.8421, 0.1484)	(0.8047, 0.2231)	(0.7625, 0.3075)	(0.7169, 0.3988)	(0.6883, 0.4559)	(0.6373, 0.5581)
2.5	3	2.15	3.36	4.18	5.30	6.61	7.58	9.53
		(0.36)	(0.58)	(0.87)	(1.26)	(1.80)	(2.34)	(3.75)
	5	1.96	2.11	2.65	3.20	3.95	4.47	5.54
		(0.20)	(0.31)	(0.50)	(0.60)	(0.83)	(0.99)	(1.49)
	10	1.00	1.69	1.97	2.03	2.43	2.75	3.33
		(0.00)	(0.46)	(0.18)	(0.18)	(0.50)	(0.52)	(0.66)
	(µ, σ)	(0.9953, 0.0325)	(0.9495, 0.1242)	(0.9177, 0.1878)	(0.8814, 0.2605)	(0.8416, 0.34)	(0.8165, 0.3903)	(0.7711, 0.4809)
2.75	3	2.05	3.12	3.76	4.50	5.27	5.75	6.70
		(0.22)	(0.49)	(0.70)	(0.87)	(1.11)	(1.29)	(1.87)
	5	1.91	2.02	2.40	2.87	3.37	3.68	4.25
		(0.28)	(0.14)	(0.49)	(0.49)	(0.60)	(0.67)	(0.87)
	10	1.00	1.41	1.90	1.99	2.10	2.33	2.74
		(0.00)	(0.49)	(0.30)	(0.11)	(0.30)	(0.47)	(0.49)
	(µ, σ)	(1.0849, 0.0274)	(1.0459, 0.1054)	(1.0186, 0.1601)	(0.987, 0.2231)	(0.9522, 0.2929)	(0.93, 0.3373)	(0.8896, 0.418)
3	3	2.01	2.96	3.45	3.98	4.49	4.78	5.30
		(0.11)	(0.42)	(0.58)	(0.67)	(0.76)	(0.85)	(1.11)
	5	1.85	2.00	2.18	2.65	2.98	3.20	3.58
		(0.36)	(0.05)	(0.39)	(0.48)	(0.45)	(0.52)	(0.62)
	10	1.00	1.16	1.78	1.97	2.00	2.04	2.31
		(0.00)	(0.37)	(0.41)	(0.18)	(0.09)	(0.21)	(0.46)
	(µ, σ)	(1.3785, 0.0155)	(1.356, 0.0606)	(1.3397, 0.0932)	(1.3205, 0.1316)	(1.2987, 0.1751)	(1.2846, 0.2035)	(1.2582, 0.2561)
4	3	2.00	2.55	2.85	2.95	3.03	3.11	3.27
		(0.02)	(0.50)	(0.36)	(0.24)	(0.26)	(0.35)	(0.46)
	5	1.37	1.99	2.00	2.00	2.11	2.24	2.48
		(0.48)	(0.09)	(0.04)	(0.08)	(0.31)	(0.43)	(0.50)
	10	1.00	1.00	1.05	1.50	1.86	1.94	1.99
		(0.00)	(0.00)	(0.23)	(0.50)	(0.34)	(0.23)	(0.11)
	(µ, σ)	(1.6045, 0.01)	(1.5898, 0.0392)	(1.5791, 0.0606)	(1.5663, 0.0862)	(1.5517, 0.1156)	(1.542, 0.1349)	(1.5238, 0.1713)
5	3	2.00	2.15	2.56	2.80	2.90	2.93	2.94
		(0.04)	(0.36)	(0.50)	(0.40)	(0.30)	(0.26)	(0.23)
	5	1.04	1.96	1.99	2.00	2.00	2.00	2.00
		(0.18)	(0.20)	(0.10)	(0.04)	(0.02)	(0.03)	(0.01)
	10	1.00	1.00	1.00	1.02	1.28	1.53	1.82
		(0.00)	(0.00)	(0.00)	(0.15)	(0.45)	(0.50)	(0.38)

		2.25	2.5	2.75	3	3.5	4	5
		ARL (SDRL)	ARL (SDRL)	ARL (SDRL)	ARL (SDRL)	ARL (SDRL)	ARL (SDRL)	ARL (SDRL)
$\alpha$	(µ, σ)	(− 3.5897, 4.4067)	(− 3.6939, 4.6151)	(− 3.7883, 4.804)	(− 3.8747, 4.9767)	(− 4.0279, 5.2832)	(− 4.1608, 5.5491)	(− 4.3833, 5.994)
Shape parameter$\gamma$								
0.25	3	1.24	1.22	1.20	1.19	1.16	1.14	1.12
		(0.47)	(0.45)	(0.44)	(0.42)	(0.40)	(0.37)	(0.34)
	5	1.07	1.06	1.05	1.04	1.03	1.03	1.02
		(0.25)	(0.24)	(0.22)	(0.20)	(0.18)	(0.16)	(0.14)
	10	1.00	1.00	1.00	1.00	1.00	1.00	1.00
		(0.04)	(0.03)	(0.03)	(0.02)	(0.02)	(0.02)	(0.01)
	(µ, σ)	(− 2.2213, 3.0564)	(− 2.3222, 3.2581)	(− 2.4142, 3.442)	(− 2.4986, 3.6109)	(− 2.6492, 3.912)	(− 2.7803, 4.1744)	(− 3.0007, 4.6151)
0.5	3	1.70	1.61	1.55	1.50	1.41	1.36	1.28
		(0.77)	(0.73)	(0.69)	(0.67)	(0.61)	(0.57)	(0.51)
	5	1.33	1.27	1.23	1.20	1.15	1.12	1.08
		(0.51)	(0.47)	(0.45)	(0.42)	(0.37)	(0.33)	(0.28)
	10	1.06	1.03	1.02	1.02	1.01	1.01	1.00
		(0.23)	(0.18)	(0.15)	(0.13)	(0.10)	(0.07)	(0.05)
	(µ, σ)	(− 1.439, 2.3026)	(− 1.5347, 2.4941)	(− 1.6228, 2.6703)	(− 1.7043, 2.8332)	(− 1.8506, 3.1258)	(− 1.9789, 3.3825)	(− 2.1959, 3.8165)
0.75	3	2.51	2.28	2.12	1.98	1.79	1.67	1.51
		(1.17)	(1.06)	(1.00)	(0.92)	(0.83)	(0.77)	(0.68)
	5	1.90	1.74	1.62	1.52	1.38	1.30	1.20
		(0.76)	(0.70)	(0.66)	(0.62)	(0.55)	(0.50)	(0.42)
	10	1.36	1.26	1.18	1.13	1.07	1.04	1.02
		(0.50)	(0.44)	(0.39)	(0.34)	(0.26)	(0.20)	(0.13)
	(µ, σ)	(− 0.9011, 1.8021)	(− 0.9905, 1.981)	(− 1.0737, 2.1474)	(− 1.1513, 2.3026)	(− 1.292, 2.584)	(− 1.4166, 2.8332)	(− 1.629, 3.2581)
1	3	4.06	3.46	3.07	2.77	2.38	2.11	1.81
		(2.07)	(1.73)	(1.49)	(1.35)	(1.15)	(1.02)	(0.85)
	5	2.97	2.54	2.27	2.07	1.78	1.60	1.39
		(1.25)	(1.04)	(0.92)	(0.84)	(0.73)	(0.66)	(0.56)
	10	2.07	1.80	1.61	1.46	1.27	1.17	1.07
		(0.71)	(0.63)	(0.58)	(0.54)	(0.46)	(0.38)	(0.25)
	(µ, σ)	(− 0.4991, 1.4446)	(− 0.5816, 1.6094)	(− 0.6592, 1.7647)	(− 0.7324, 1.911)	(− 0.8665, 2.1793)	(− 0.9866, 2.4195)	(− 1.1935, 2.8332)
1.25	3	8.54	6.15	4.92	4.13	3.25	2.75	2.21
		(5.37)	(3.55)	(2.70)	(2.18)	(1.64)	(1.35)	(1.08)
	5	5.79	4.28	3.47	2.95	2.35	2.04	1.65
		(3.04)	(2.04)	(1.55)	(1.26)	(0.98)	(0.84)	(0.68)
	10	3.68	2.83	2.35	2.04	1.66	1.43	1.19
		(1.49)	(1.03)	(0.83)	(0.71)	(0.61)	(0.53)	(0.40)
	(µ, σ)	(− 0.3645, 1.3291)	(− 0.444, 1.4883)	(− 0.5193, 1.6389)	(− 0.5906, 1.7814)	(− 0.7219, 2.044)	(− 0.84, 2.2803)	(− 1.0444, 2.689)
1.35	3	13.65	8.56	6.31	5.07	3.76	3.09	2.41
		(9.83)	(5.44)	(3.71)	(2.82)	(1.95)	(1.56)	(1.17)
	5	8.80	5.70	4.29	3.52	2.68	2.25	1.79
		(5.39)	(3.01)	(2.07)	(1.60)	(1.13)	(0.94)	(0.74)
	10	5.32	3.61	2.81	2.36	1.85	1.57	1.27
		(2.53)	(1.47)	(1.05)	(0.83)	(0.66)	(0.58)	(0.45)
	(µ, σ)	(− 0.0259, 1.0517)	(− 0.0969, 1.1937)	(− 0.1652, 1.3303)	(− 0.2306, 1.4612)	(− 0.353, 1.706)	(− 0.4648, 1.9295)	(− 0.6611, 2.3221)
1.649	3	217.11	48.06	19.79	11.50	6.44	4.64	3.17
		(213.31)	(43.37)	(15.72)	(8.16)	(3.88)	(2.56)	(1.62)
	5	188.82	28.98	11.78	7.27	4.27	3.18	2.27
		(185.59)	(24.50)	(8.03)	(4.16)	(2.10)	(1.42)	(0.96)
	10	138.73	15.02	6.57	4.31	2.74	2.13	1.58
		(133.27)	(10.72)	(3.48)	(1.94)	(1.03)	(0.76)	(0.59)
	(µ, σ)	(0.0718, 0.9757)	(0.0036, 1.1121)	(− 0.0624, 1.244)	(− 0.1258, 1.3709)	(− 0.2451, 1.6094)	(− 0.3546, 1.8285)	(− 0.548, 2.2152)
1.75	3	328.27	114.74	35.75	17.38	8.07	5.40	3.49
		(321.85)	(110.82)	(30.97)	(13.48)	(5.22)	(3.13)	(1.81)
	5	269.93	81.23	21.10	10.33	5.17	3.65	2.48
		(262.06)	(76.60)	(16.59)	(6.82)	(2.71)	(1.70)	(1.05)
	10	188.35	44.25	10.73	5.79	3.22	2.38	1.70
		(181.33)	(38.86)	(6.93)	(2.98)	(1.30)	(0.86)	(0.62)
	(µ, σ)	(0.2842, 0.8179)	(0.2227, 0.941)	(0.1624, 1.0615)	(0.1038, 1.1787)	(− 0.0078, 1.4018)	(− 0.1116, 1.6094)	(− 0.2974, 1.981)
2	3	66.32	97.33	95.39	52.33	16.15	8.59	4.62
		(59.41)	(91.17)	(90.24)	(47.42)	(12.47)	(5.73)	(2.58)
	5	29.23	54.68	63.89	32.44	9.54	5.38	3.13
		(22.18)	(48.25)	(58.59)	(27.66)	(6.19)	(2.85)	(1.41)
	10	11.91	24.01	34.65	16.86	5.33	3.28	2.07
		(6.56)	(18.21)	(29.10)	(12.32)	(2.67)	(1.34)	(0.75)
	(µ, σ)	(0.4644, 0.6931)	(0.4089, 0.804)	(0.354, 0.9138)	(0.3001, 1.0217)	(0.1961, 1.2296)	(0.0981, 1.4256)	(− 0.0798, 1.7814)
2.25	3	20.11	27.91	35.85	41.39	29.64	14.46	6.28
		(13.07)	(21.08)	(29.54)	(35.66)	(25.12)	(10.85)	(3.87)
	5	9.85	14.11	19.33	24.54	18.26	8.65	4.06
		(4.50)	(8.38)	(13.80)	(19.07)	(13.84)	(5.38)	(2.00)
	10	5.15	7.02	9.53	12.41	9.86	4.92	2.58
		(1.53)	(2.87)	(5.10)	(7.89)	(5.96)	(2.36)	(0.97)
	(µ, σ)	(0.6196, 0.5933)	(0.5697, 0.6931)	(0.5198, 0.793)	(0.4703, 0.892)	(0.3737, 1.0852)	(0.2814, 1.2698)	(0.1116, 1.6094)
2.5	3	10.28	12.82	15.62	18.63	23.63	19.77	8.51
		(4.44)	(6.85)	(9.84)	(13.11)	(18.58)	(15.57)	(5.64)
	5	5.95	7.27	8.95	10.81	14.85	12.42	5.39
		(1.75)	(2.73)	(4.26)	(6.10)	(10.31)	(8.54)	(2.91)
	10	3.54	4.21	4.99	5.92	8.26	7.12	3.28
		(0.75)	(1.10)	(1.65)	(2.41)	(4.59)	(3.82)	(1.34)
	(µ, σ)	(0.7554, 0.5125)	(0.7104, 0.6024)	(0.665, 0.6931)	(0.6196, 0.7839)	(0.53, 0.9631)	(0.4434, 1.1365)	(0.2816, 1.46)
2.75	3	7.04	8.09	9.34	10.65	13.79	16.31	11.03
		(2.13)	(2.98)	(4.22)	(5.60)	(8.92)	(11.78)	(7.77)
	5	4.46	5.09	5.81	6.61	8.57	10.47	6.97
		(0.97)	(1.35)	(1.92)	(2.61)	(4.53)	(6.52)	(3.97)
	10	2.86	3.18	3.56	3.97	5.01	6.23	4.22
		(0.49)	(0.63)	(0.85)	(1.14)	(2.00)	(3.07)	(1.86)
	(µ, σ)	(0.8755, 0.4463)	(0.8349, 0.5274)	(0.7937, 0.6099)	(0.752, 0.6931)	(0.669, 0.8591)	(0.5878, 1.0217)	(0.434, 1.3291)
3	3	5.50	6.06	6.70	7.43	9.11	11.01	11.72
		(1.26)	(1.67)	(2.21)	(2.91)	(4.66)	(6.66)	(8.06)
	5	3.70	4.05	4.44	4.87	5.86	7.10	7.82
		(0.66)	(0.85)	(1.10)	(1.44)	(2.33)	(3.53)	(4.52)
	10	2.43	2.67	2.88	3.10	3.63	4.33	4.82
		(0.50)	(0.51)	(0.56)	(0.70)	(1.09)	(1.64)	(2.15)
	(µ, σ)	(1.2488, 0.2749)	(1.2214, 0.3298)	(1.1928, 0.3871)	(1.1632, 0.4463)	(1.102, 0.5685)	(1.0397, 0.6931)	(0.9158, 0.941)
4	3	3.33	3.47	3.61	3.75	4.07	4.46	5.37
		(0.48)	(0.53)	(0.59)	(0.68)	(0.93)	(1.27)	(2.16)
	5	2.54	2.67	2.74	2.81	2.98	3.21	3.81
		(0.50)	(0.47)	(0.45)	(0.45)	(0.56)	(0.75)	(1.27)
	10	1.99	2.00	2.00	2.00	2.06	2.19	2.55
		(0.08)	(0.05)	(0.03)	(0.07)	(0.24)	(0.41)	(0.67)
	(µ, σ)	(1.5172, 0.1844)	(1.4979, 0.2231)	(1.4773, 0.2643)	(1.4557, 0.3075)	(1.41, 0.3988)	(1.3621, 0.4947)	(1.2629, 0.6931)
5	3	2.95	2.94	2.94	2.95	3.00	3.12	3.46
		(0.23)	(0.23)	(0.24)	(0.26)	(0.39)	(0.53)	(0.85)
	5	2.00	2.00	2.01	2.03	2.15	2.30	2.57
		(0.01)	(0.03)	(0.08)	(0.18)	(0.36)	(0.46)	(0.57)
	10	1.87	1.94	1.96	1.96	1.96	1.96	1.96
		(0.34)	(0.25)	(0.21)	(0.20)	(0.20)	(0.20)	(0.25)

**Table 3:** parameters for the proposed control chart.

	$\left( {\alpha_{0} ,\beta_{0} } \right)$	L	$\lambda$	ARL_0_
1	(1.65, 2.16)	3.345	0.3	370
2	(1.65, 2.16)	3.252	0.2	370

## Main findings

1. By holding the scale constant *α* = 1.649, and varying the shape parameter, a diverse array of outcomes can be attained. The alterations in mean and standard deviation correspond directly to the magnitude and direction of the shape parameter adjustment. Specifically, for shape parameter values exceeding 2.16, even with an ARL_0_ = 370 indicating control, the normal process can manifest fluctuations in both its mean and standard deviation. For *α* = 1.64, we observed the change in transformed mean and standard deviation with the change in shape parameter. Because of this modification, the mean and standard deviation will move together, resulting in lower values of ARL_1_. For instance, when considering increased sample sizes, the ARL_1_ falls significantly. Specifically, when the sample size is 3, the ARL_1_ decreases from 217 to 3.17. Similarly, for a sample size of 5, the ARL_1_ decreases from 188 to 2.27. Furthermore, when the sample size is 10, the ARL_1_ decreases from 138 to 1.58. In the case of a downward shift ($\gamma$ < 2.16). The changes in the ARL_1_ decreased trend from 214.92 to 2.83 for a sample size of 3, from 92.93 to 2.00 for sample size 5 and from 30.96 to 0.17 for sample size 10.
2. When altering the scale parameter while maintaining the shape parameter constant, a downward shift is noticeable. This adjustment leads to a more significant displacement in the corresponding standard parameters compared to the upward displacement. The magnitude of the shift in the Lognormal scale parameter is directly proportional to the magnitude of the change in a normal parameter. For instance, when *α* = 5.00, the shift in the mean and standard deviation is determined to be 1.5238 and 0.1713, respectively.
3. When holding the scale and shape parameters constant and manipulating the sample size, it is seen that the ARL_1_ and the SDRL_1_ drop as the sample size increases. This implies that if the producer is able to handle a big sample size, it is possible to promptly identify an out-of-control process by raising the sample size while maintaining the same scale and shape parameter. For Example in Table 2 for *L*(3, 2.5) with *n* = 3 the ARL_1_ and SDRL_1_ are (6.06, 1.67), for *n* = 5 (4.05, 0.85), and for *n* = 10 (2.67, 0.51) respectively. So we can easily observe the decreasing trend in ARLs and SDRLs.
4. When the scale and shape parameters (2, 2.25) are kept constant and the smoothing constant is varied, it is observed that decreasing the smoothing constant leads to a drop in both the ARL_1_ and the SDRL_1_ values.

## Application to engine oil data

**Figure 1:**
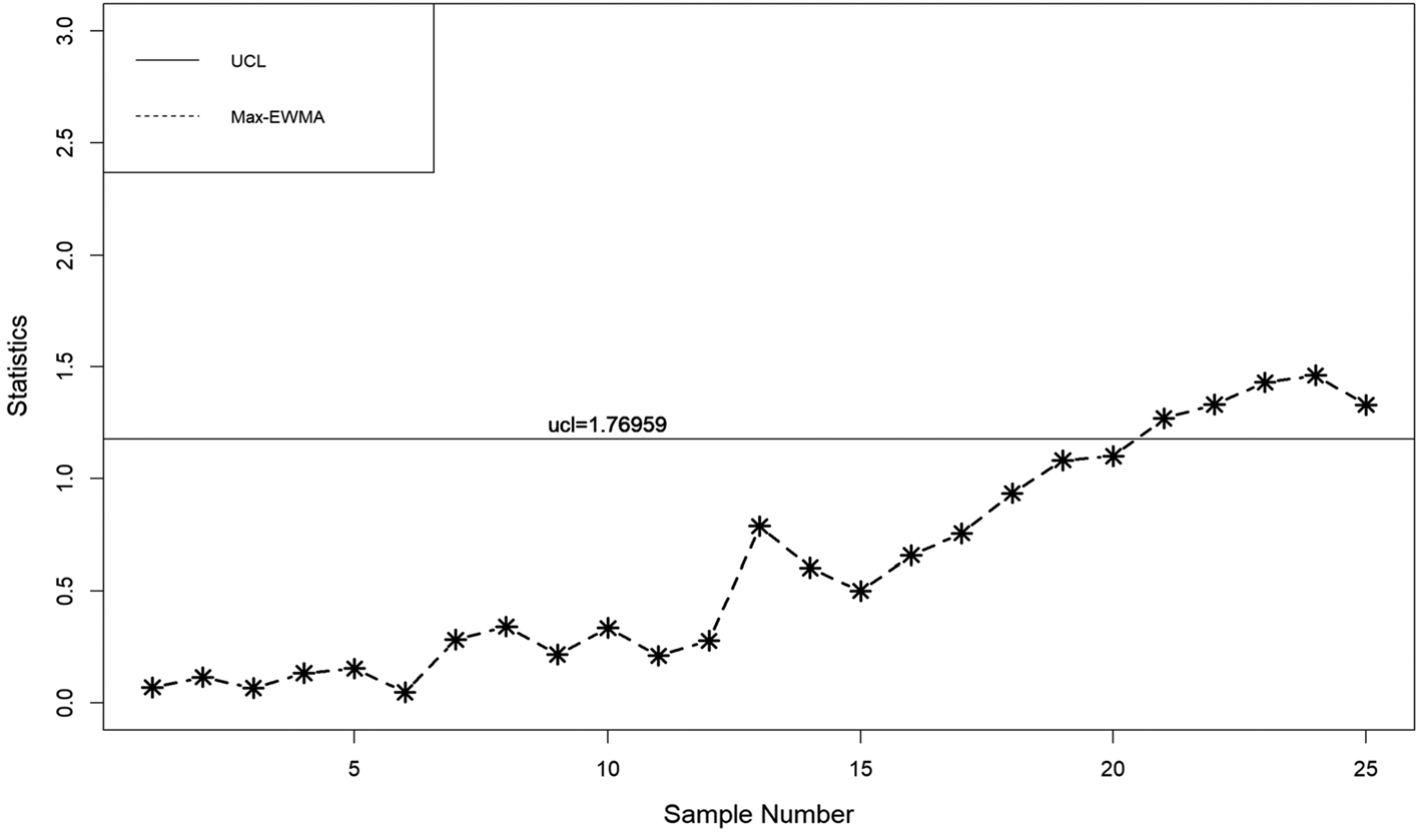
λ = 0.2, L = 3.985, n = 3

**Figure 2:**
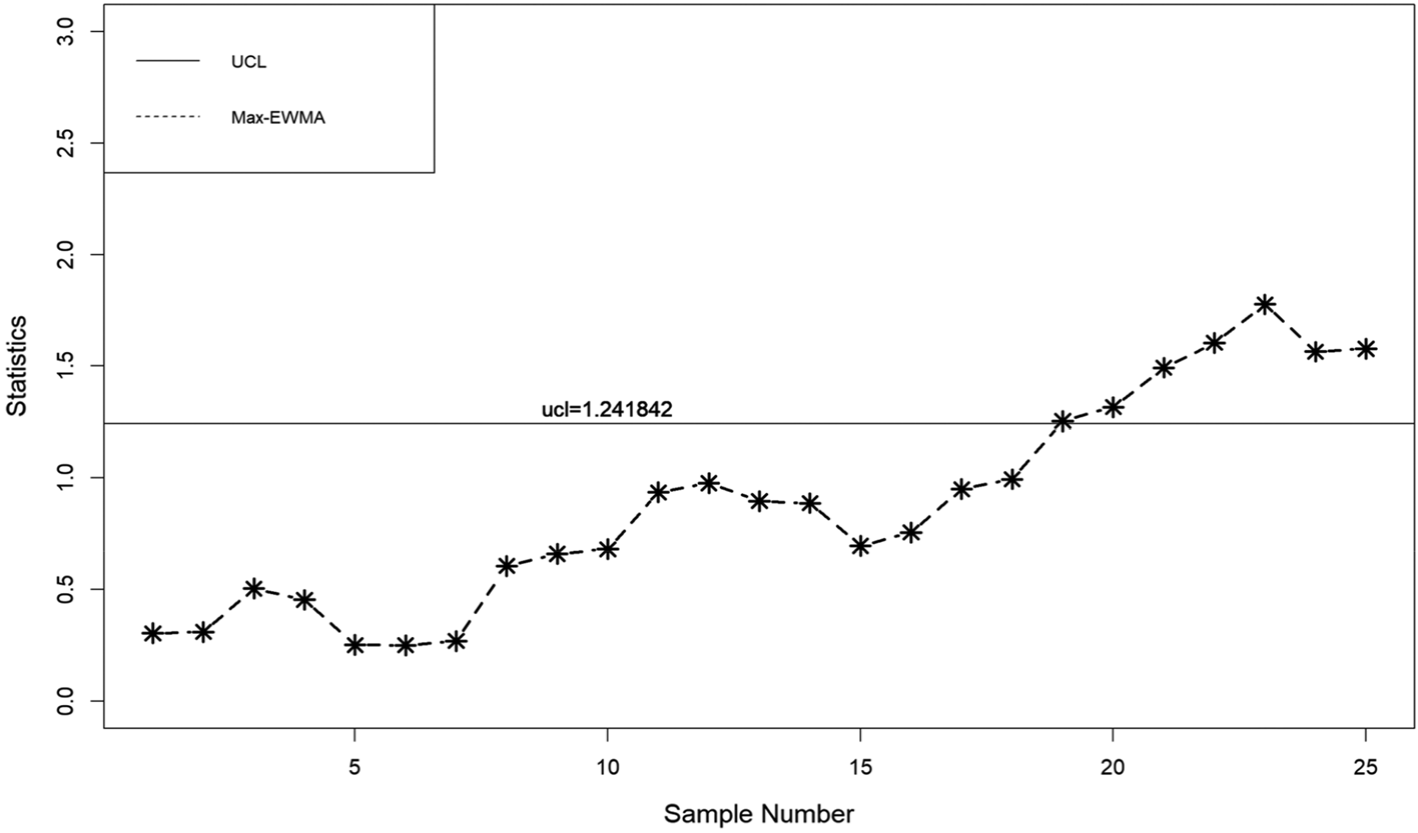
λ = 0.2, L = 4.3084, n = 5

**Figure 3:**
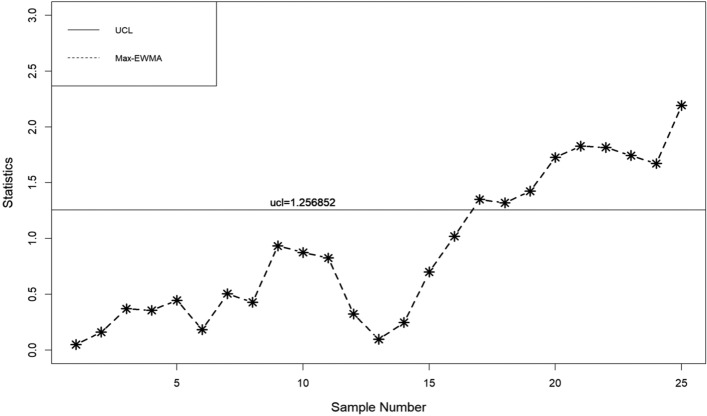
λ = 0.2, L = 4.3831 n = 7

In this part, a practical example is presented to demonstrate the application of the suggested lognormal Max-EWMA chart. The Test Monitoring Center provides the ASTM D7320 Reference Oil Data. In order to evaluate the quality of engine oil, particularly in new automobiles, quality engineers need to measure the percent viscosity increase (PVIS). The data set is taken from Huang et al.^19^ where they determined that the probability volume integral signature adheres to a lognormal distribution. The concept of distribution refers to the way in which resources, goods, or services are allocated. There exists a specific type of reference oil, namely Ref Oil 434, which possesses certain quality characteristics that require attention. The experiment was conducted. In order to establish a reference for oils, a total of 50 samples, each consisting of 10 units, were gathered and subsequently employed for analysis. The data about the Ref Oil 434 are shown in Table 4. The reader may consult Huang et al.^19^ for further details on the dataset. In the case of Ref Oil 434, as depicted in Figs. 1, 2, and 3, it is observed that there are instances of out-of-control samples occurring at the 21st position for sample sizes of *n* = 3 and 19th position for sample size of *n* = 5. Additionally, sample 17 is found to be outside the control limits with a sample size of *n* = 7, as indicated by the proposed control chart for λ = 0.2. Hence, it may be inferred that as the sample size increases, the efficiency of detecting instances of being out of control also increases.

**Table 4:** PVIS data for Ref Oil 434.

No.	*x*_*i*1_	*x*_*i*2_	*x*_*i*3_	*x*_*i*4_	*x*_*i*5_	*x*_*i*6_	*x*_*i*7_	*x*_*i*8_	*x*_*i*9_	*x*_*i*10_
1	54.2	103.7	160.7	381.5	108.4	870.8	114.1	120.6	67.4	185.7
2	90.7	411.3	160.7	106.5	114.1	114.1	69.2	106.3	119.1	133
3	106.5	83.7	107.4	92.2	104	110.9	69.2	35.4	297.9	73.9
4	297.9	89.9	35.4	76.1	252.2	54.2	155.1	381.5	373.1	94.2
5	108.4	613.6	137.6	76.1	110.8	411.3	169	83.7	373.1	110.8
6	110.9	356	54.2	107.4	83.7	494.5	151.9	196.5	303.3	185.7
7	307.6	88.6	97.8	94.2	101.5	129.6	76.1	90.8	106.5	103.4
8	119.1	93	145.9	667.9	76.9	94.9	80.7	356	141.2	75.5
9	83.5	127.6	93	83.5	72.3	82.8	53.2	83.1	85	86.8
10	108.9	66.4	300.5	134	115.4	117.5	103.7	103.7	83.1	300.5
11	76.1	133.3	303.3	89.9	185.7	373.1	70	80.7	60.9	69.2
12	94.2	252.2	114.1	103.7	106.5	99.2	122.7	75.5	127.6	133.3
13	105.8	101.5	53.2	70	151.9	89.9	160.7	196.5	97.1	249.5
14	88.9	73.9	151.9	151.3	307.6	303.3	356	300.5	46.2	107.4
15	104	205	90.8	667.9	83.1	307.6	252.2	88.9	613.6	106.3
16	613.6	101.5	53.6	117.5	303.6	81.6	94.2	106.3	97.1	110.2
17	185.7	90.8	119.1	324.4	74.8	86.7	108.4	90.7	324.4	205
18	146.3	105.8	90.8	115.4	62.8	160.7	107.4	303.3	83.1	83.5
19	97.8	46.2	667.9	185.7	83.1	83.5	80.7	160.7	124.8	155.1
20	97.1	141.2	76.1	169	151.9	494.5	70	94.2	92.2	83.1
21	129.6	88.9	411.3	90.7	151.9	137.6	226.3	106.5	307.6	94.8
22	66.4	281.6	86.8	179.1	124.8	94.9	83.1	118.9	93.7	103.7
23	160.7	83.5	92.2	105.8	146.3	300.5	151.9	494.5	124.8	86.8
24	80.7	117.5	411.3	179.1	120.6	137.6	76.1	196.5	145.9	88.9
25	93.7	134	185.7	870.8	69.2	307.6	67.4	76.1	185.7	76.1
26	89.9	80.7	97.1	145.9	81.6	119.1	145.9	118.9	76.1	85
27	90.8	115.4	97.1	92.2	83.5	88.6	94.2	133.3	82.8	205
28	99.2	70	281.6	60.9	70	147.2	99.2	303.6	55.4	114.1
29	226.3	107.2	226.3	76.9	297.9	70	106.3	303.6	97.8	107.2
30	226.3	46.2	83.1	93.7	35.4	185.7	870.8	83.5	90.8	110.2
31	179.1	373.1	300.5	300.5	114.1	53.6	106.3	122.7	356	101.5
32	108.9	108.1	83.7	155.1	185.7	373.1	147.2	94.8	88.8	107.4
33	72.3	53.2	46.2	137.6	124.8	179.1	69.7	76.1	89.9	97.8
34	72.3	86.8	60.9	107.2	117.5	94.8	76.1	94.9	53.2	160.7
35	307	89.9	151.3	373.1	46.2	70	281.6	104	86.8	90.8
36	67.4	88.8	83.7	54.2	196.5	127.6	373.1	69.7	70	61.1
37	133	115.4	667.9	172	94.9	124.8	107.4	85.9	108.4	185.7
38	88.6	127.6	110.2	103.7	147.2	147.2	303.3	72.3	86.7	303.6
39	300.5	108.4	110.8	115.4	92.2	141.2	103.7	119.1	88.8	93.7
40	613.6	97.1	134	97.8	205	103.4	108.4	108.1	103.7	110.2
41	93	90.8	297.9	110.2	124.8	151.9	870.8	179.1	252.2	85
42	137.6	356	80.7	106.3	75.5	179.1	118.9	82.8	120.6	411.3
43	46.2	381.5	62.8	411.3	81.6	86.7	88.8	108.9	90.7	97.1
44	128.7	76.1	108.9	160.7	99.2	110.2	72.3	62.8	97.8	53.6
45	73.9	81.6	107.2	103.7	85.9	35.4	117.5	151.3	90.7	133.3
46	90.7	101.5	297.9	85.9	107.2	667.9	148.2	108.1	97.1	373.1
47	494.5	103.7	196.5	146.3	160.7	134	53.2	119.1	133	94.6
48	297.9	99.2	133.3	103.7	101.5	97.8	494.5	249.5	129.6	103.4
49	83.1	46.2	103.7	110.9	86.7	35.4	103.7	226.3	169	146.3
50	105.8	69.7	108.4	196.5	303.3	86.7	411.3	75.5	249.5	110.8

**Figure 4:**
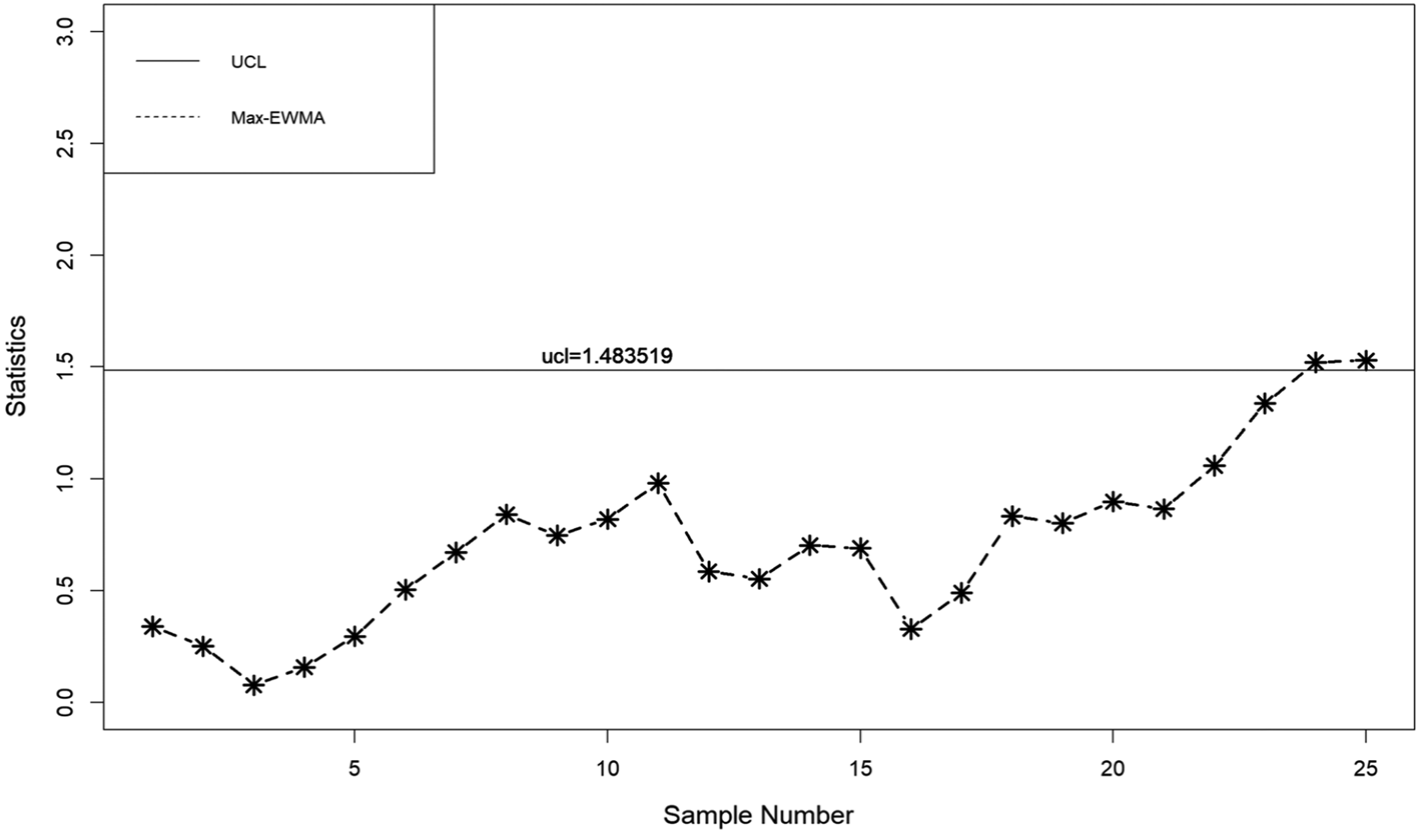
λ = 0.3, L = 3.9865 n = 3

**Figure 5:**
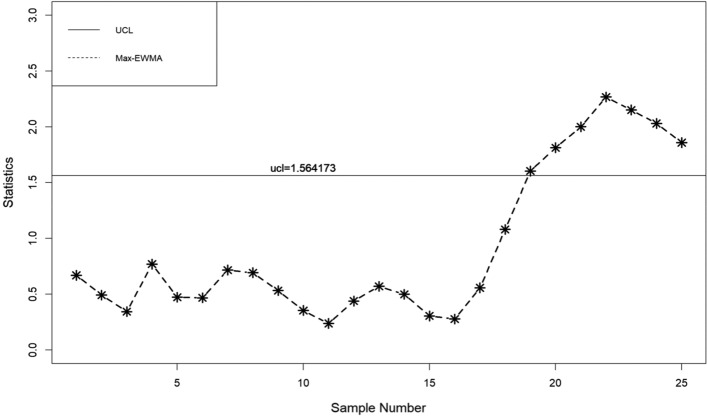
λ = 0.3, L = 4.305 n = 5

**Figure 6:**
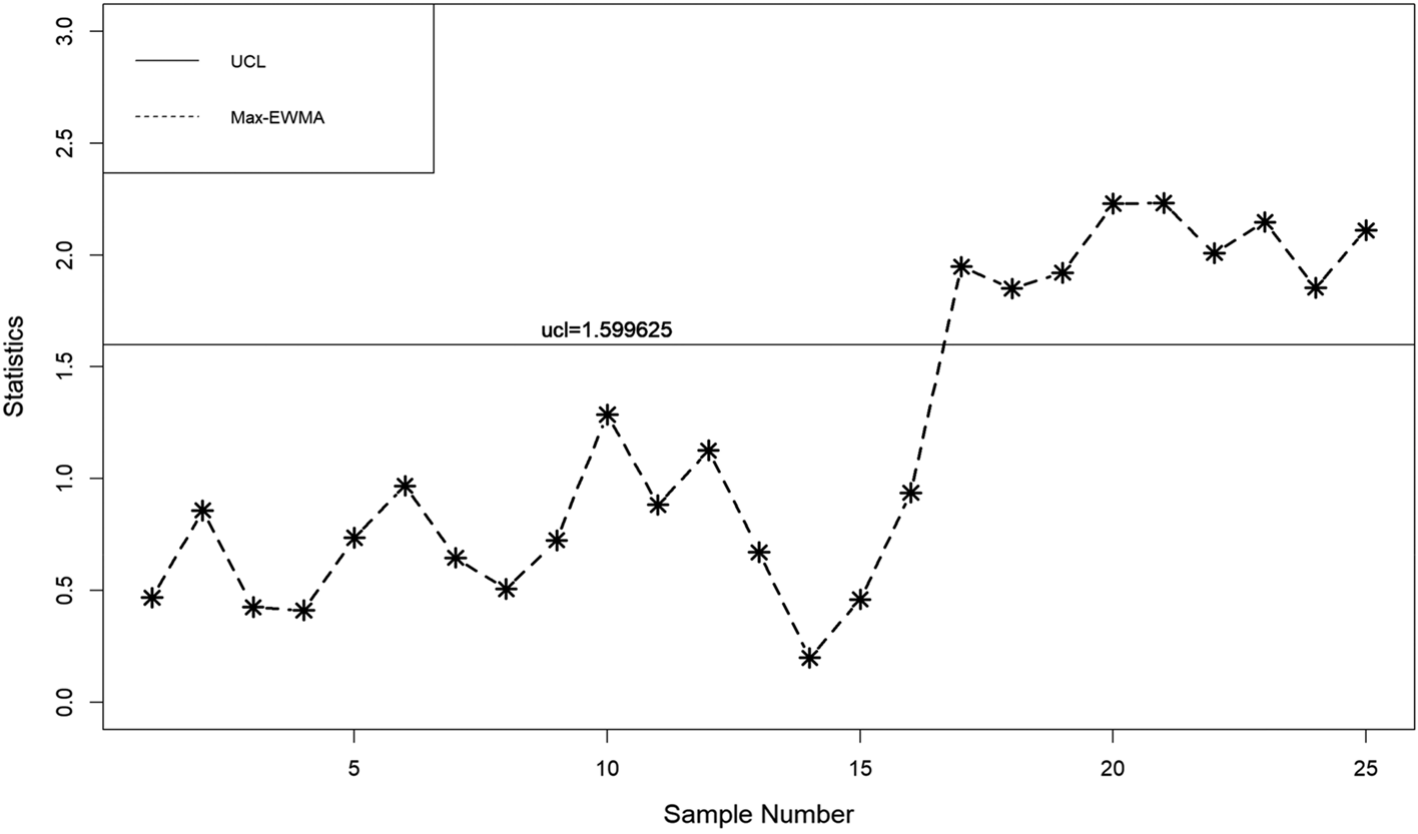
λ = 0.3, L = 4.445 n = 7

When λ = 0.2, as illustrated in Figs. 4, 5, and 6, it is evident that there are occurrences of out-of-control samples at the 24th position for a sample size of *n* = 3 and at the 19th position for a sample size of *n* = 5. Furthermore, it has been seen that sample 17, with a sample size of *n* = 7, falls outside the control limits as depicted by the suggested control chart. Therefore, it can be deduced that as the size of the sample increases, the ability to detect instances of being out of control likewise improves.

## Conclusion

The establishment of Max-EWMA control charts for concurrently tracking the lognormal distribution’s mean and standard deviation is covered in this paper. According to the simulation experiments, the Max-EWMA charts perform well in cases where the underlying lognormal distribution is more skewed. An increase in the lognormal shape parameter (λ > 2.16) results in a little change in both the mean and standard deviation. As the value of λ grows, there is a decrease observed in both the mean and standard deviation shifts. A decrease in the lognormal shape parameter (λ < 2.16) is associated with reductions in both the mean and standard deviation. As the value of λ declines, there is an increase in the shift observed in the standard deviation of the normal parameter. This increase in shift leads to a reduction in the ARL_1_ values from 217 to 3.17. The inverse response function is employed for the purpose of transformation.

## Data Availability

The datasets used and/or analyzed during the current study are available from the corresponding author upon reasonable request.
